# The Association Between Goal Setting and Weight Loss: Prospective Analysis of a Community Weight Loss Program

**DOI:** 10.2196/43869

**Published:** 2023-07-05

**Authors:** Gina M Wren, Dimitrios A Koutoukidis, Jadine Scragg, Michael Whitman, Susan Jebb

**Affiliations:** 1 Nuffield Department of Primary Care Health Sciences University of Oxford Oxford United Kingdom; 2 NIHR Oxford Biomedical Research Centre Oxford University Hospitals NHS Foundation Trust Oxford United Kingdom; 3 Second Nature London United Kingdom

**Keywords:** obesity, overweight, weight loss, goals, motivation, mobile app, mobile health, mHealth, behavior change, mobile phone

## Abstract

**Background:**

Goal setting aids health-related behavior changes; however, the influence of different types of goals on weight loss remains unclear.

**Objective:**

We aimed to investigate the association of 3 aspects of goal setting with weight and program dropout over a 24-week period.

**Methods:**

This study was a prospective longitudinal analysis of participants in a 12-week digital behavioral weight loss program. Weight and engagement data for eligible participants (N=36,794) were extracted from the database. Eligible participants were adults in the United Kingdom who had enrolled in the program, had a BMI ≥25 kg/m^2^, and a weight reading recorded at baseline. Three aspects of goal setting were self-reported at enrollment: weight loss motivation (appearance, health, fitness, or self-efficacy), overall goal preference (low, medium, or high), and percentage weight loss goal (<5%, 5%-10%, or >10%). Weight was measured at 4, 12, and 24 weeks. Mixed models for repeated measures were used to explore the association between goals and weight across the 24-week period. To measure sustained weight change, the primary outcome was weight at 24 weeks. We explored dropout rates over the 24-week period by goal and whether engagement mediated the association between goals and weight loss.

**Results:**

Of the 36,794 participants (mean 46.7, SD 11.1 years; 33,902/36,794, 92.14% female) included in the cohort, 13.09% (n=4818) reported weight at 24 weeks. Most participants set goals of 5%-10% weight loss (23,629/36,794, 64.22%), but setting goals for >10% was associated with greater weight loss (mean difference 5.21 kg, 95% CI 5.01-5.41; *P*<.001). There was no difference between goals of 5%-10% and <5% (mean difference 0.59 kg, 95% CI 0.00-1.18; *P*=.05). Appearance was the most prevalent motivational factor (14,736/36,794, 40.05%), but health and fitness were associated with greater weight losses (mean difference health vs appearance 1.40 kg, 95% CI 1.15-1.65; *P*<.001 and mean difference fitness vs appearance 0.38 kg, 95% CI 0.05-0.70; *P*=.03). Goal preference had no association with weight. Engagement was an independent predictor of weight loss but not a mediator of the effect of goal setting. At 24 weeks, those who set goals of >10% were less likely to drop out compared with 5%-10% goals (odds ratio [OR] 0.40, 95% CI 0.38-0.42; *P*<.001); those who liked to set overall high goals were more likely to drop out compared with medium goals (OR 1.20, 95% CI 1.11-1.29; *P*<.001); and those motivated by fitness or health were less likely to drop out compared with appearance (OR 0.92, 95% CI 0.85-0.995; *P*=.04 and OR 0.84, 95% CI 0.78-0.89; *P*<.001, respectively).

**Conclusions:**

Setting higher weight loss goals and being motivated by health or fitness were associated with greater weight loss and lower likelihood of dropout. Randomized trials for setting these types of goals are required to confirm causality.

## Introduction

### Background

Goal setting is an important motivational factor underlying changes in health behavior [1]. Goal setting theory is based on the principle that conscious goals direct attention and action, and that conscious behavior is regulated by an individual’s goals [2,3]. Setting a weight loss goal has been shown to lead to greater weight loss than not setting a goal [4], prescribing a higher physical activity goal has been shown to lead to greater weight loss [5], and interventions that incorporate both goal setting and self-monitoring have been found to be more effective at promoting health behaviors than interventions without these techniques [6].

The study of goal setting in the context of weight loss is particularly relevant, given the disparity between what physicians and patients who are overweight and obese consider a *realistic* goal. A 5%-10% reduction in weight is widely accepted as clinically meaningful because of associated improvements in cardiometabolic risk factors and is recommended by current guidelines as a weight loss target for people who are overweight or obese [7-9]. However, overweight and obese individuals regularly set weight loss goals 3 to 4 times (22%-34% weight reduction) greater than what is recommended [10-13].

There is some uncertainty regarding the best strategy for incorporating goals into behavioral programs for weight loss. On the basis of the findings from the broader goal setting literature, when goals are too ambitious, individuals experience impaired performance, which often leads to abandonment of the goal [14,15]. In the context of obesity research, retrospective studies have found that setting larger weight loss goals may result in poor weight loss maintenance [16,17]. Similarly, setting larger weight loss goals was associated with higher rates of attrition from therapy and smaller reductions in BMI after 12 months of treatment for obesity [12,18,19]. Meanwhile, other studies found either no relationship or a modest positive relationship between setting larger weight loss goals and achieved weight losses [11,20-23] and no relationship between failure to meet weight loss goals and attrition [22,24]. Previous studies of weight loss goals had small sample sizes and it is possible that the importance of goal setting differs across different behavioral programs.

It may also be important to consider how the perceived magnitude of an individual’s goal relates to weight outcomes. Failure to meet weight loss goals that are perceived as realistic may have a greater impact on long-term success than failure to meet ambitious goals, which individuals may recognize are less attainable. However, studies have reported mixed results in terms of outcomes. Women who are overweight and obese have been reported to lose more weight when their pretreatment weight loss expectations are high [25]. Similarly, participants who were instructed to set *realistic* lower goals lost less weight than those instructed to set *unrealistic* higher goals, which were 1.5 times higher [26]. No association was found between *goal* weights and outcomes, but *dream* weights (which participants perceived as less likely to achieve than *goal* weights) were associated with greater weight loss at 18 months [11]. Conversely, the observation that *dream* BMI, but not *acceptable* or *expected* BMI, was negatively associated with BMI change at 1 year shows that goals that are perceived as high may have a negative impact on the weight loss achieved [18]. However, this observation may be driven by the greater attrition rate among participants with higher weight loss expectations [18].

Motivation is a key psychosocial factor that influences weight loss success [27,28]. Frequently reported motivational factors for losing weight include improving physical appearance and health [18,24,29,30] and wanting to feel better about oneself [30,31]. Intrinsic motivations, such as an interest in exercise, predict long-term weight maintenance [32], whereas extrinsic motivations, such as appearance, have been associated with smaller weight losses [27,29,33]. Behavioral weight loss programs typically focus on increasing an individual’s level of motivation; however, there is little evidence on how the type of the initial motivation affects subsequent weight change. A better understanding of the initial motivations for considering weight loss programs is important as a lack of participant motivation is commonly used as an explanation for intervention failure or poor intervention outcomes [34]. Examining a participant’s initial reasons for weight loss could help to predict intervention outcomes.

### Objectives

The primary aim of this study was to investigate the association of 3 different aspects of goal setting (weight loss motivation, overall goal preference, and percentage weight loss goal) with weight change over a 24-week period. The secondary aims were to investigate whether engagement mediated the association between goals and weight loss and to explore the association between goals and participant dropout.

## Methods

### Study Design and Participants

This prospective longitudinal study used data collected by Second Nature. Second Nature provides a digitally delivered behavioral program that aims to support people to increase their physical activity and create sustainable healthy eating behaviors.

Data for eligible participants were retrieved from the Second Nature database in March 2022. The researchers who conducted the analysis received a deidentified data set. All participants were adults in the United Kingdom, aged ≥18 years, and had a BMI ≥25 kg/m^2^ at program entry. To be eligible for inclusion in the analysis, participants must have paid to participate in the 12-week behavioral program seeking support for weight loss, recorded a baseline weight reading, and completed the bespoke health questionnaire during the enrollment process. All participants consented to the use of their anonymized data for medical research purposes by accepting the privacy policy as part of the sign-up process (Multimedia Appendix 1).

### Program Description

**Figure 1:**
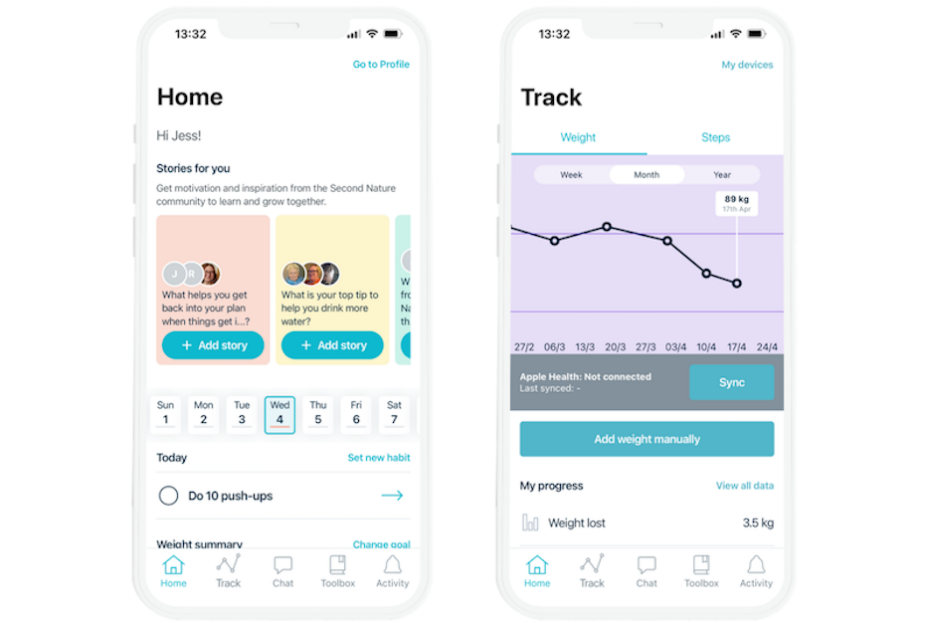
Example Second Nature program content.

The 12-week behavioral change program consisted of mentoring from a health coach (registered dietitian or nutritionist), peer group support, educational articles, and activity tracking technology. The program was accessed via a smartphone or a web-based app (Figure 1). Before the start of the program, each participant received an instructional handbook and recipe book and they could optionally pay extra to receive wireless weighing scales.

The behavioral component of the intervention has been informed by behavioral frameworks aiming to promote successful behavior change, including those outlined in the behavior change wheel [35]. A number of behavior change techniques were used within the various functions of the app and incorporated into the health coaching support, including self-monitoring (behavior and outcome), goal setting (behavior and outcome), feedback (behavior and outcome), social support (practical), and instruction on how to perform the behavior [36].

Participants were allocated a health coach who delivered personalized support via an SMS text messaging service within the app. The messaging support was provided both privately and within a group chat of up to 14 other participants to provide social accountability and motivation. Educational information, delivered through plain text and videos, could be accessed by participants through the app. The educational information covered topics focused on healthy eating, physical activity, stress management, and sleep. Participants were able to record and view their weight and step readings within the app. These could also be viewed by the health coach who encouraged participants to engage with the app and monitor their progress against their goals. The frequency of recording weight readings varied among the participants and was informed by individual choices. Health coaches were notified when participants had low engagement (defined as <10 interactions with the app in the previous week) to contact them and encourage participation.

### Data Collection

At baseline, each participant answered a series of questions in a bespoke health questionnaire, including their goals for the program. Participants also self-reported their sex, height, age, presence of type 2 diabetes or prediabetes, and home postcode (which was used to calculate socioeconomic deprivation using the Index of Multiple Deprivation [IMD] [37]).

Weight data were either automatically collected using the Bluetooth weighing scales provided at the start of the program or could be manually input into the app. Having a weight reading at baseline was part of the criteria for inclusion in the analysis so the data set included complete weight data at baseline. However, for validation purposes, baseline weight readings were only retrieved from the database if they ranged between 40 kg and 200 kg. Weight data were collected at 3 further follow-up time points: 4 weeks, 12 weeks, and 24 weeks. A single weight reading was extracted for each time point by searching within a specified period (3-5 weeks for 4 weeks, 10-14 weeks for 12 weeks, and 20-28 weeks for 24 weeks) and the reading closest to the midpoint of each period was extracted. A validation algorithm, which took into account the previous reading and time since this reading was registered, was used for readings at each collection time point to only accept readings within an expected range. These validation processes were put in place to exclude anomalous readings and ensure that consistent and objective readings were extracted for analysis.

Engagement data captured participants’ interaction with the 3 main components of the smartphone app: learn, track, and support. Learn interactions were defined as the total number of articles read. Track interactions were defined as the number of times a participant viewed or had a recorded weight or steps reading. Support interactions were defined as the number of messages sent or received in either a private or group chat. Engagement was measured as the cumulative total number of interactions with these 3 components of the app at 3 time points: 0-4 weeks, 0-12 weeks, and 0-24 weeks. The exact cutoff time point for each period was defined based on the date of the extracted single weight reading.

### Measures

#### Exposures

The following three aspects of goal setting were self-reported in the bespoke health questionnaire, which was completed as part of the enrollment process and before commencement of the program: (1) weight loss motivation—participants selected their primary reason for weight loss, categorized as appearance, health, fitness, or self-efficacy; (2) goal preference*—*participants selected whether they normally like to set low, medium, or high goals; and (3) percentage weight loss goal—percentage of initial body weight that participants were aiming to lose, categorized as <5%, 5%-10%, or >10%. The questionnaire was completed independently with no additional instructions.

#### Mediators

Total engagement, defined as the cumulative number of interactions, was considered as a mediating variable.

#### Outcomes

To measure sustained weight change, the primary outcome was weight at 24 weeks. The secondary outcome was dropout, defined as program cancelation up to and including each of the time points (4, 12, and 24 weeks).

### Statistical Analysis

A prespecified analysis plan was published on the Open Science Framework [38]. Descriptive statistics were used to examine baseline characteristics of the study population, and 2-tailed *t* tests or chi-squared tests were used to compare differences in characteristics. A cross-tabulation analysis was used to explore the relationship between the different aspects of goal setting. We also explored the association between baseline characteristics and each aspect of goal setting.

Three independent mixed models for repeated measures were used to explore the association between the 3 aspects of goal setting and the dependent outcome variable, weight, over a 24-week period. A between-subjects factor of goal, a within-subjects factor of week, and the interaction between week and goal were included as fixed effects. Participant was included as a random effect to account for the repeated weight measures on the same participant at 4, 12, and 24 weeks. Further models adjusted for prespecified covariates (sex, age, IMD decile, and type 2 diabetes or prediabetes) as fixed effects. Diabetes was included as a covariate in addition to demographic characteristics as it is a weight-related condition that often affects weight loss and can also influence motivation to complete and submit weight readings in weight loss programs [39].

In a mixed effects model, missing data can be accommodated through maximum likelihood estimation, which allows for the inclusion of all available data from participants. For this analysis, a sequential testing approach was used, as follows:

1. Model 1—random effect: participant ID, fixed effects: goal and week
2. Model 2—random effect: participant ID, fixed effect: goal and week, interaction term: goal × week
3. Model 3—random effect: participant ID, fixed effect: goal and week, interaction term: goal × week, adjusted for sex (male or female), age (years, continuous), IMD decile (factor), and type 2 diabetes or prediabetes (yes or no)

The random effects term in the mixed effects model indirectly took into account differences in baseline weight. Model fit was compared using the *R*^2^ statistic and a *P* value was calculated using a likelihood ratio. The final model, adjusting for all covariates (model 3), produced the best fitting model; therefore, this is the only model presented hereafter.

A multivariable logistic regression was used to explore the association between goals and the likelihood of dropout of the program at each time point. All models were adjusted for sex, age, IMD decile, type 2 diabetes or prediabetes, and baseline weight.

### Sensitivity Analyses

A sensitivity analysis was conducted by repeating the analysis of the primary outcome using completers only (ie, participants with complete data at all time points) to confirm the validity of the findings and to illustrate the pattern of weight change in the same individuals over time. As there were 8403 missing IMD values and 3054 missing goal preference values, a sensitivity analysis using the missing indicator method was also conducted.

### Mediation Analysis

Mediation analysis explored whether total engagement mediated the association between goals and weight [40]. Step 1 of the mediation analysis was the primary analysis. Step 2 of the mediation analysis was a mixed effects model on the same sample, testing whether there was a significant association between goals and engagement while adjusting for all covariates. For step 3, the mixed effects model of step 1 was repeated with additional adjustment for engagement as a predictor. An engagement variable was considered as a mediator if engagement significantly predicted weight and the effect of goals was attenuated with adjustment for the engagement variable. The indirect effect and proportion of total effect mediated were calculated.

All analyses were conducted using R (version 4.1.3; R Foundation for Statistical Computing) with the Integrated Development Environment R Studio. All reported *P* values are for 2-sided tests, with effects considered statistically significant at *P*<.05.

### Ethical Considerations

This study was reviewed by the University of Oxford Medical Sciences Interdivisional Research Ethics Committee (reference R84327/RE001). As the study only involved the use of previously collected, anonymized, and non–National Health Service data that cannot be traced back to identifiable individuals, it was confirmed as exempt from ethical review.

## Results

### Baseline Characteristics

The mean age of the total sample was 46.7 (SD 11.1) years and mean baseline BMI was 34 (SD 6.46) kg/m^2^, and 92.14% (33,902/36,794) of the participants were female (Table 1).

Most participants were motivated to lose weight for appearance reasons (14,736/36,794, 40.05%), said they preferred to set medium goals (21,963/36,794, 59.69%), and set a weight loss goal of 5%-10% of the initial body weight (23,629/36,794, 64.22%; Tables S1-S3 in Multimedia Appendix 2). More participants (641/2370, 27.05%) who said they preferred low goals set a percentage weight loss goal of <5%. There was no clear relationship between the other aspects of goal setting.

Older participants tended to be motivated by health and fitness, male participants tended to be motivated less by appearance and self-efficacy than female participants, and those who had a higher baseline BMI were motivated more by health reasons (Table S4 in Multimedia Appendix 2). Those motivated by fitness set the lowest percentage weight loss goals, and those motivated by health set the highest percentage weight loss goals. The distribution of IMD deciles was different between the motivation groups; in general, more people from higher IMD deciles selected appearance as their reason for weight loss and more people from lower IMD deciles selected fitness or self-efficacy. Participants who preferred to set higher goals were younger, a greater proportion were male, had higher baseline BMI (compared with those who preferred to set lower goals), and set higher percentage weight loss goals (Table S5 in Multimedia Appendix 2). Participants who set higher percentage weight loss goals were younger, had a greater proportion who were male, and had a higher baseline BMI (Table S6 in Multimedia Appendix 2).

**Table 1:** Baseline characteristics of total sample, participants with complete weight data (n=3193), and participants with incomplete weight data.

			Total (N=36,794)		Complete data (n=3193)		Incomplete data (n=33,601)		*P* value^a^
Age (years), mean (SD)			46.7 (11.1)		48.7 (10.5)		46.5 (11.1)		<.001
**Sex, n (%)**									.16
	Male	2892 (7.86)		272 (8.52)		2620 (7.79)			
	Female	33,902 (92.14)		2921 (91.48)		30,981 (92.2)			
BMI (kg/m^2^), mean (SD)			34.0 (6.46)		33.5 (6.39)		34.1 (6.46)		<.001
**IMD^b^ decile, n (%)**									.07
	1-3	4379 (11.9)		357 (11.18)		4022 (11.97)			
	4-7	12,091 (32.86)		1054 (33.01)		11,037 (32.85)			
	8-10	11,921 (32.39)		1103 (34.54)		10,818 (32.19)			
	Missing	8403 (22.84)		679 (21.27)		7724 (22.99)			

^a^*P* values were calculated using 2-tailed *t* tests for continuous variables or chi-squared tests for categorical variables to compare differences between participants with complete weight data and participants with incomplete weight data. Complete data were defined as having weight readings recorded at all time points (ie, at 4, 12, and 24 weeks).

^b^IMD: Index of Multiple Deprivation.

### Retention and Data Completeness

Among those who remained in the program, weight data were incomplete. Of the 36,794 participants in the cohort, 42% (n=15,455) had weight data available at 4 weeks, 20.73% (n=7627) at 12 weeks, and 13.09% (n=4818) at 24 weeks, whereas 8.68% (n=3193) had weight readings recorded at all time points. Examination of the participants with complete weight data at all time points, compared with participants with at least one missing weight reading, found no difference between sex or IMD decile (Table 1). Although statistically significant differences were observed for age and baseline BMI (both *P*<.001), these differences were not clinically meaningful.

**Figure 2:**
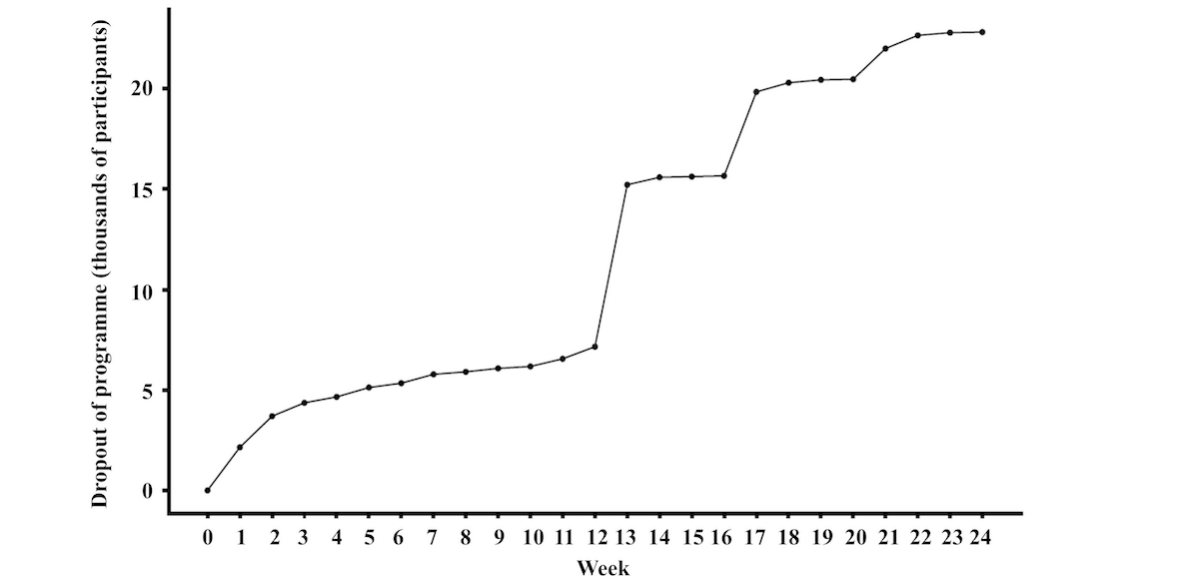
Percentage dropout of program over 24 weeks. The total number of included participants at week 0 was 36,794.

Cumulative dropout rates of the program at 4, 12, and 24 weeks were 13.9%, 41.4%, and 62%, respectively. Figure 2 shows the dropout rates from 0 to 24 weeks. There was some dropout in the first 2 weeks of the program, followed by a steady rate of dropout week-on-week until the end of the program at 12 weeks. There was a large amount of dropout at the end of the 12-week program, and then up to 24 weeks dropout occurred on a monthly basis.

### Association Between Goal Setting and Weight Change Over a 24-Week Period

In the whole cohort, the mean unadjusted weight change at 4, 12, and 24 weeks was −4.16 (SD 2.94) kg, −5.93 (SD 4.31) kg, and −6.40 (SD 5.97) kg, respectively.

**Figure 3:**
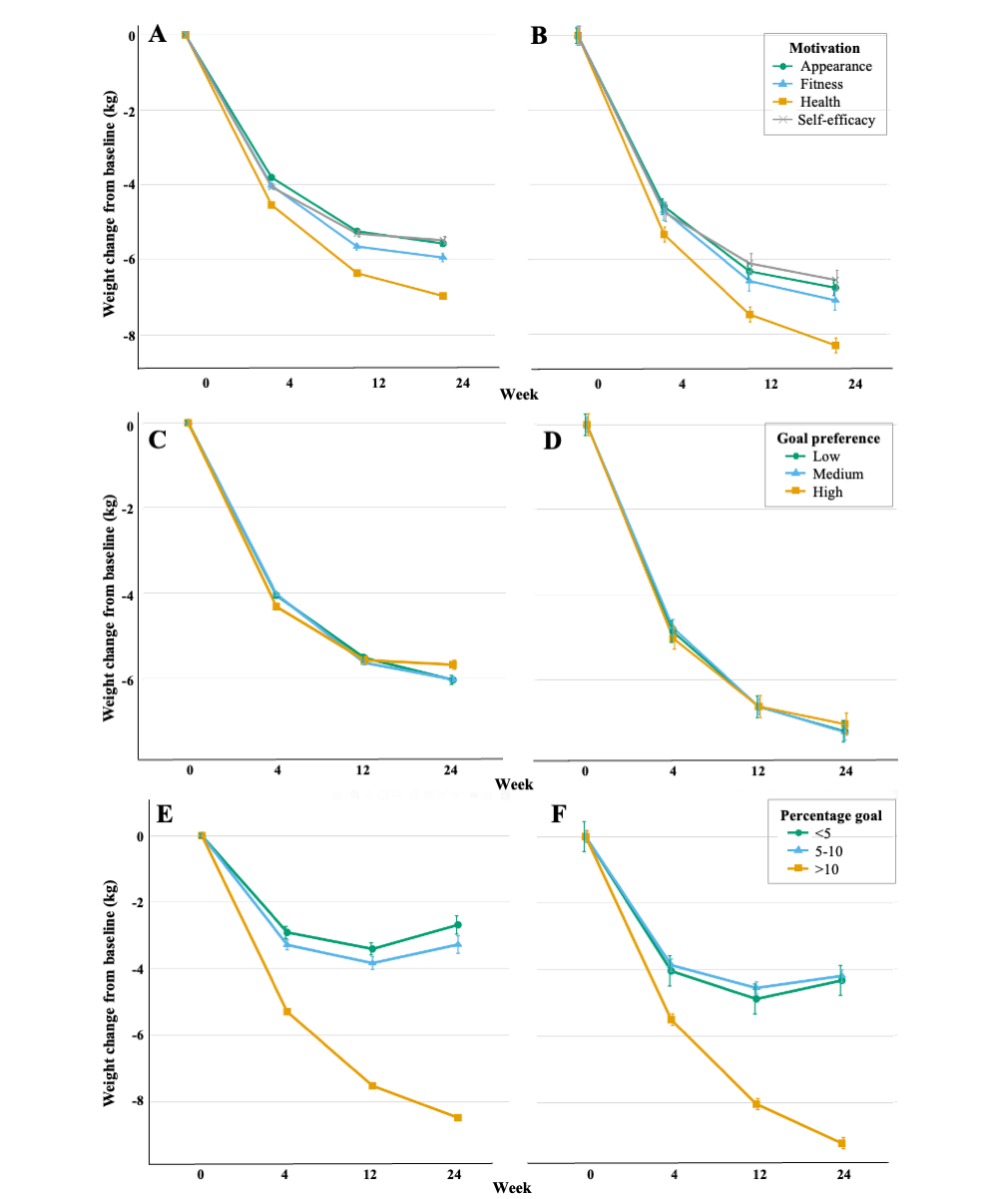
Mean adjusted weight change by (A) weight loss motivation using all available data (n=28,391), (B) weight loss motivation for completers (n=2514), (C) goal preference using all available data (n=26,158), (D) goal preference for completers (n=2194), (E) percentage weight loss goal using all available data (n=28,391), and (F) percentage weight loss goal for completers (n=2514). Weight change (kg) at 4, 12, and 24 weeks were calculated from mixed effects models, adjusted for age, sex, Index of Multiple Deprivation, and type 2 or prediabetes. Values represent mean (SE).

At baseline, those motivated for fitness, health, and self-efficacy reasons weighed 6.1 kg, 14.8 kg, and 10.1 kg more than those motivated by appearance, respectively. At 24 weeks, compared with appearance, those motivated for health reasons lost 1.40 kg more (95% CI 1.15-1.65; *P*<.001), and those motivated for fitness reasons lost 0.38 kg more than appearance (95% CI 0.05-0.70; *P*=.03; Figure 3A and Table S7 in Multimedia Appendix 2). A sensitivity analysis using completers only also found that those motivated by health lost more weight at all time points, but there was no difference between appearance and fitness (Figure 3B and Table S7 in Multimedia Appendix 2).

Those who preferred low goals weighed 3.60 kg (95% CI 2.98-4.22; *P*<.001) more at baseline than those who preferred medium goals. There was no difference between medium and high goals. Compared with those who preferred medium goals, high goal preference was associated with greater weight loss at 4 weeks, but lesser weight loss at 24 weeks. At 24 weeks, those who preferred high goals lost 0.34 kg less (95% CI 0.05-0.64; *P*=.02) than those who preferred medium goals (Figure 3C and Table S8 in Multimedia Appendix 2). A sensitivity analysis using completers only did not replicate these results and found that there was no difference between the different goal preferences at 4, 12, or 24 weeks (Figure 3D and Table S8 in Multimedia Appendix 2).

At baseline, those who set goals of <5% weighed 7.99 kg less (95% CI 7.09-8.90; *P*<.001) and those who set goals of >10% weighed 0.99 kg less (95% CI 0.51-1.47; *P*<.001), compared with goals of 5%-10%. Those who set goals of >10% lost significantly more weight at all time points compared with those who set goals of 5%-10%. At 24 weeks, those who set goals of >10% lost 5.21 kg more (95% CI 5.01-5.41; *P*<.001) compared with those who set goals of 5%-10% (Figure 3E and Table S9 in Multimedia Appendix 2). Furthermore, those who set goals of >10% continued to lose weight after the program ended up until 24 weeks, whereas those who set goals of <10% regained some of the weight lost after the program ended. A sensitivity analysis using completers only found a similar pattern of weight change over 24 weeks for each percentage category (Figure 3F and Table S9 in Multimedia Appendix 2).

Further sensitivity analyses using the missing indicator method (Tables S10-S12 in Multimedia Appendix 2) also showed similar results for each aspect of goal setting.

### Association Between Goal Setting and Weight Change as Mediated by Engagement

Tracking was the most frequent form of engagement at every time point, but all types of engagement declined over time (Multimedia Appendix 3).

There was no evidence that engagement mediated the difference in weight loss between the motivation categories (Table S13 in Multimedia Appendix 2) or the percentage weight loss goal categories (Table S14 in Multimedia Appendix 2).

### Association Between Goal Setting and Dropout of Program

There was a lower likelihood of dropout for those motivated by health compared with those motivated by appearance at all time points (Table 2). At 24 weeks, those motivated by fitness reasons were less likely to drop out compared with appearance. There was a lower likelihood of dropout for those who preferred medium goals compared with those who preferred high or low goals at 4 and 12 weeks. There was also a lower likelihood of dropout for those who preferred medium goals at 24 weeks compared with those who preferred high goals. Finally, those who set lower percentage weight loss goals were more likely to drop out at 4, 12, and 24 weeks.

**Table 2:** Association between goals and likelihood of dropout of program at 4, 12, and 24 weeks^a^.

Variables		Dropout at 4 weeks		Dropout at 12 weeks		Dropout at 24 weeks	
		OR^b^ (95% CI)	*P* value	OR (95% CI)	*P* value	OR (95% CI)	*P* value
**Motivation (ref: appearance)**							
	Fitness	0.94 (0.85-1.03)	.18	0.93 (0.86-1.00)	.054	0.92 (0.85-0.995)	.04
	Health	0.90 (0.83-0.98)	.01	0.88 (0.83-0.94)	<.001	0.84 (0.78-0.89)	<.001
	Self-efficacy	1.09 (1-1.18)	.04	1.04 (0.97-1.11)	.29	1.05 (0.98-1.14)	.17
**Goal preference (ref: medium)**							
	Low	1.20 (1.10-1.30)	<.001	1.17 (1.09-1.25)	<.001	1.07 (1-1.16)	.06
	High	1.13 (1.04-1.22)	.002	1.17 (1.09-1.24)	<.001	1.20 (1.11-1.29)	<.001
**Percentage weight loss goal (ref: 5%-10%)**							
	<5%	2.71 (2.45-3.01)	<.001	1.60 (1.44-1.77)	<.001	1.32 (1.17-1.5)	<.001
	>10%	0.40 (0.37-0.44)	<.001	0.46 (0.44-0.49)	<.001	0.40 (0.38-0.42)	<.001

^a^Models were adjusted for sex, age, Index of Multiple Deprivation decile, type 2 diabetes, or prediabetes.

^b^OR: odds ratio.

## Discussion

### Principal Findings

The program led to clinically meaningful weight loss of 6.40 kg (equivalent to 6.8%) at 24 weeks among the participants who continued to weigh themselves. Differences in demographic characteristics or goal setting were small. Health and fitness motivations were associated with greater weight losses and lower likelihood of dropout compared with appearance at 24 weeks. Setting weight loss goals of >10% was associated with an average 5.21 kg greater weight loss than setting the more common 5%-10% goal, and an average 60% lower odds of dropout at 24 weeks. There was no clear association between goal preference and weight loss.

### Comparison With Prior Work

In other studies on weight loss motivating factors, the primary factor was to improve health, whereas appearance had a low prevalence [12,29]. These differences could be because of the recruitment strategy and resultant selection bias; patients recruited from medical centers in a research setting may be more likely to give health as their reason for weight loss, compared with this analysis of data in a community setting where participants were self-funding program attendance. However, consistent with previous research, we found that health motivation was associated with greater weight loss than appearance [29,33]. This could be explained by self-determination theory and goal contents [41]. Goal contents are distinguished by the extent to which they fulfill basic psychological needs. Intrinsic goals (eg, to improve health) are more closely related to the fulfillment of psychological needs, whereas extrinsic goals (eg, to improve appearance) are not essential to well-being and personal development. Research has shown that extrinsic goals provide motivation in the short term, whereas intrinsic goals are more beneficial for long-term results [42]. Although health or fitness motivators cannot be easily categorized as intrinsic or extrinsic, the results of this study suggest that the underlying motivational reason may be more important than increasing overall motivation. Weight loss motivation may differ according to demographic characteristics and may explain previously observed differences in outcomes [30,43].

The finding that participants who set larger weight loss goals lost more weight at 24 weeks challenges current UK clinical guidelines that encourage a weight loss goal of 5%-10% [7]. In fact, this study supports previous findings demonstrating that setting a higher weight loss goal is associated with greater weight loss at 12 months than lesser weight loss goals [23], and a systematic review that reports goal difficulty as one of the main factors that makes goal setting effective [44]. This study also found that participants who set goals of >10% continued to lose weight after the program ended, whereas those who set goals of <10% regained some weight for up to 24 weeks. For some people, setting a larger weight loss goal may be more motivating, and it has been suggested that higher goals are more self-relevant and provide a sense of direction and purpose [45]. Higher goals have also been associated with greater effort in the weight loss attempt [21], as predicted by goal setting theory, which suggests that goals have an energizing function, causing greater effort directed toward more challenging goals [2]. These findings suggest that recommendations on setting realistic weight loss goals should be reconsidered.

Goal preference had no clear association with weight loss. As the primary outcome was weight, we were only able to consider participants who submitted weight readings. It is possible that those who stopped weighing themselves or dropped out were unsuccessful in their weight loss attempts. This temporal sequence of events has been shown previously, whereby users tend to gain weight and reduce their weight loss efforts before ceasing weight tracking [46]. Thus, the lack of association with goal preference could be driven by differences in attrition, as those who liked to set high goals were 1.2 times more likely to drop out by 24 weeks (*P*<.001). The association between higher weight loss expectations and greater attrition has also been shown previously [18]. It is possible that when goals are perceived as too ambitious, individuals experience impaired performance, which discourages a person’s belief in their ability to control their weight, leading to abandonment of weight management behaviors [14,15,47]. Alternatively, the bespoke health questionnaire, completed as part of the enrollment process, may not have accurately measured goal preference, particularly as this is a subjective measure, as shown by a lack of correlation between the goal preference and percentage weight loss goal variables.

Overall, the mediation analysis found that participants who engaged more with program components tended to lose more weight, as reported previously [48-50], but this was independent of the type of goal set. Therefore, engagement can be considered an independent predictor of weight loss but not a mediator of the association between goals and weight. Maintaining engagement and retaining participants over time are key to the success of digital weight loss interventions. The modification of goal setting via pretreatment recommendations may be a means to maintain engagement, which is worth further exploration.

### Strengths and Limitations

To date, no study in a community setting has explored the association between goals and weight loss on this scale in detail, with 3 different aspects of goal setting reported. The use of this data set allows for greater confidence than other studies in terms of the robustness and generalizability of the findings. The strengths of this study include the large sample size, the preregistration of the analysis plan, and prospective design. Although the population sample was predominantly female, this is broadly representative of enrollment in most private weight management services [51,52].

The limitations of this study largely reflect the challenges associated with analyses of a population in an uncontrolled, community setting. The observational nature means that causal relationships cannot be determined, and randomized controlled trials are required to confirm these results. There was a large proportion of missing weight readings as participants were not actively encouraged to weigh themselves or engage with the program after the initial program ended at 12 weeks. Many participants canceled their subscription after completing the main 12-week program, as shown in Figure 2, which is reflected in the availability of weight data at 24 weeks. Similar proportions of missing weight data have been reported previously for self-funded participants enrolled in the same program [39]. We aimed to mitigate the issue of missing weight readings by using mixed models for repeated measures, allowing use of all available data with weight readings assumed missing at random. The sensitivity analyses did not lead to different conclusions. However, participants who continue to register weight readings are likely to be more motivated, and to have lost weight, resulting in self-selection bias. Although the exposure groups did not significantly differ by demographic characteristics, we cannot rule out the possibility that they may have differed by other factors that were not measured here, such as ethnicity. Further research is needed to extrapolate these findings to more demographically diverse populations, particularly male participants and those from more socioeconomically deprived backgrounds. The sample only included participants who were paying to participate in the program, meaning that this population was likely to be more self-motivated to lose weight. Payment for participation may have influenced participants’ commitment, engagement, and their initial motivation for starting the program. As such, the generalizability of these findings to nonpaying populations remains unknown.

### Implications and Further Research

Current guidelines recommend weight loss of 5%-10%, but encouraging individuals to set larger goals may lead to improved weight loss outcomes and better retention in weight loss programs. Future randomized controlled trials testing the effect of pretreatment counseling to set larger weight loss goals on program success are warranted. Investigating whether there is an upper limit for the association between higher weight loss goals and achieved weight losses would also be relevant for future research.

Although further research cannot realistically change participants’ primary motivation for weight loss, 2 randomized controlled trials have shown that weight loss interventions tailored to specific types of weight loss motivation achieve greater weight loss compared with controls [53,54]. Motivating factors for weight loss may be used to predict who is most likely to be successful and who may need more support, particularly as a lack of participant motivation is commonly used to explain poor intervention outcomes [34]. Without more complete weight data, it was not possible to establish the long-term outcomes of all the participants in this cohort. Further research is needed to determine ways to maintain engagement, as retaining participants over time is likely critical for the optimal effectiveness of digital weight loss programs.

### Conclusions

Setting larger weight loss goals and being motivated for health or fitness reasons were associated with greater weight loss and a lower likelihood of dropout. Randomized trials testing the effects of setting these types of goals are required to confirm causality.

## Appendix

Multimedia Appendix 1 Second Nature privacy policy.

Multimedia Appendix 2 Cross-tabulation analyses, baseline characteristics by goal aspect, full results of mixed effects models, and mediation analyses by engagement.

Multimedia Appendix 3 Mean engagement with program components.

## Abbreviations

IMD: Index of Multiple Deprivation
OR: odds ratio

## Data Availability

The participants of this study did not provide written consent for their data to be shared publicly; thus, supporting data are not available.
