# Supplementary material for: The Association Between Goal Setting and Weight Loss: Prospective Analysis of a Community Weight Loss Program
Source: J Med Internet Res. 2023 Jul 5;25:e43869. doi: 10.2196/43869 (PMC10357317; doi:10.2196/43869)
Supplement: Multimedia Appendix 1 [file jmir_v25i1e43869_app1.docx]

**MULTIMEDIA APPENDIX – PRIVACY POLICY**

**The Association Between Goal Setting and Weight Loss: Prospective Analysis of a Community Weight Loss Program**

This is a Multimedia Appendix to a full manuscript published in the J Med Internet Res. For full copyright and citation information see <http://dx.doi.org/10.2196/jmir.43869>

**Privacy Policy**

Second Nature Healthy Habits Ltd, trading under the name Second Nature, ("we") are committed to protecting and respecting your privacy. We are registered with the UK Information Commissioner's Office as a Data Controller (Reg No. ZA148098), and have in place a comprehensive Company data protection policy and code of practice.

This Privacy Policy ("Policy") (together with our Terms, which can be accessed at https://www.secondnature.io/terms, and any other documents referred to on it) sets out the basis on which any personal information we collect from you, or that you provide to us, will be processed by us and how you can get access to this information. If you are located in the European Economic Area (“EEA”) or the United Kingdom (UK), “personal information” means any information relating to an identified or identifiable individual. Please review it carefully.

1. **Purpose of this Policy**

Second Nature provides you (the "User") with access to the online and mobile services including but not limited to, secondnature.io and all associated subdomains (the "Website"), the Second Nature mobile application (the "App"), and any provided healthcare tracking technology, collectively the "System". Our privacy policy is designed in accordance with numerous national and international regulation frameworks, including (but not limited to) the General Data Protection Regulation (“GDPR”), the UK Data Protection Act 2018 and the UK General Data Protection Regulation (“UK GDPR”).

1. **What personal information do we hold and how we get it**

We may collect and process personal information provided by filling in forms on the Website or App, including personal information provided during completion of surveys and other online tools, posting of comments in the Community or requesting further services, and when you report a problem with our System. If you contact us, we may also keep a record of that correspondence. Second Nature also collects and processes personal information with the health tracking technology provided as part of the System, such as wireless weighing scales (which track your weight) and activity trackers (which track your steps and sleep).

Throughout your use of the System we may collect personal information such as: personal demographics information (including but not limited to first and last names, date of birth or age, address, email, phone number); lifestyle or health data (referred as “special category data” and includes height, weight, body mass index, ethnicity, smoking status); other personal health profile information and details of your visits to the System and the resources that you access (including, but not limited to, traffic data, location data, weblogs, other communication data, and the resources that you access).

Your personal information (steps per day and weight) may also be collected via Apple HealthKit or Google Fit upon installing our iOS and Android apps. This consent will be explained and obtained from you within the app and you may revoke this access at any point within your phone's operating system settings.

Your personal information, including your health data (referred as “special category data” and includes height, weight, HBA1C level, BP level, cholesterol level, PAM score) may also be provided to us by an electronic patient record, referral or through a secure online platform in order to refer you to the System, e.g. provided through your GP, local NHS service or our partner REED. If this is the case, the relevant party will ask for your explicit consent .

We collect as well technical information and analytics from you concerning your use of the System, including but not limited to pages visited, links clicked, non-sensitive text entered, mouse movements, system and operating system type and version, browser or app version, time zone setting and usage of our iPhone and Android apps.

1. **IP addresses and cookies**

We may collect personal information about your device, including where available your IP address, operating system, browser type and screen size for use in system administration, to tailor your experience of the System, provide you with customer support and to report aggregate information internally.

For the same reason, we may obtain personal information about your usage of the System by using a cookie file which is stored on the hard drive of your device. Cookies help us to give you a smooth user experience, improve the System and deliver a better and more personalized service. They enable us to: recognize you when you return to our site; maintain personal information you have entered (e.g. during completion of a survey); speed up your searches; estimate our audience size and usage pattern; store information about your preferences; and allow us to customize our site according to your individual interests.

Both Second Nature and our third-party vendors, including Google, may use first-party cookies (such as the Google Analytics cookie) to inform, optimize, and serve ads based on your past visits to the Website on sites across the Internet (also known as 'remarketing'). If you would like to opt out of this you can do so via your Google Ads Preferences Manager.

Below is an overview of the types of cookies we and third parties may use to collect information.

- — Strictly necessary cookies. Some cookies are strictly necessary to make the services available to you. We cannot provide you with the services without this type of cookies.
- — Functional cookies. These are used to recognize you when you return to the services. This enables us to adapt our content for you, and remember your preferences (for example, your choice of language or region).
- — Analytical or Performance cookies. We also use cookies for website and app analytics purposes in order to operate, maintain and improve our services. We may use our own analytics cookies or use third party analytics providers such as Facebook, FOSPHA, HotJar, Metabase, Mixpanel, Twitter, Taboola and VWO to collect and process certain analytics data on our behalf. These providers may also collect information about your use of other websites, apps, and online resources. You can opt out of … without affecting how you visit our services by going to ….

Where required by applicable law, we obtain your consent to use cookies.

You may refuse to accept cookies by changing the settings on your device to prevent cookies from being set. However, if you select this setting you may be unable to access certain parts of the System. Unless you have adjusted your browser setting so that it will refuse cookies, our system will issue cookies when you visit the Website and App.

1. **How we use your personal information**

Second Nature is dedicated to maintaining the privacy and integrity of your personal information. As such, we have policies and procedures and other safeguards to help protect your personal information from improper use and disclosure.

We collect and use your personal information to deliver our contract to you. We may collect and use your personal information, including your special category data, only if you have given us your specific consent.

We may use and disclose your personal information and special category data for our internal operations, which include administration, planning and various activities that assess and improve the quality and cost effectiveness of the service that we deliver to you. Examples are using information about you to improve quality of the service, satisfaction surveys, de-identifying personal information, customer services and internal training. We may use and disclose your personal information to contact you as a reminder to interact with, or complete tasks relating to your use of the System.

We use also automated decision-making to allocate you to a group of users before starting the use of our service.

We follow a Minimum Necessary Access Policy, so any required disclosure of your identifiable personal information is minimized. The following categories describe different ways that we use your personal information within Second Nature and disclose your personal information to persons and entities outside of Second Nature. We have not listed every use or disclosure within the categories below, but all permitted uses and disclosures will fall within one of the following categories. In addition, there are some uses and disclosures that may require your specific authorization.

How much personal information is used or disclosed without your written permission will vary depending, for example, on the intended purpose of the use or disclosure.

- — Disclosure at your request: We may disclose personal information relating to your use of the System when requested by you. This disclosure at your request may require written authorization by you.
- — Payment: We do not store your credit/debit card details; they are processed directly by a third party processor (for example, Stripe or Braintree) that will store all payment information and transaction details. We will only retain details of transactions on secure servers and we will not retain your credit or debit card information.
- — Operations: We may use and disclose your personal information for our internal operations, which include administration, planning and various activities that assess and improve the quality and cost effectiveness of the service that we deliver to you. Examples are using information about you to improve quality of the service, satisfaction surveys, de-identifying personal information, customer services and internal training.
- — Reminders and notifications: We may use and disclose your personal information to contact you as a reminder to interact with, or complete tasks relating to your use of the System.
- — Third party service providers:
  - — We may share personal information with third-party service providers we have hired to provide services on our behalf, including those who act as data processors on our behalf. Those data processors are subject to privacy and security obligations consistent with our privacy policy and with the current data protection regulation and framework. They can only use and process the personal information in the ways specified by us. These service providers include (but are not limited to) Amazon Web Services which provides the Second Nature app and website, analytics and search engine providers (including but not limited to Mixpanel, HotJar, Intercom) which assist us in improving Second Nature app or website and your user experience, help us to collect information on your use of the System, and to assist you in case of an issue.
  - — We may share anonymised information with third-party service providers who assist us in our marketing and advertising activities or in the improvement of Second Nature app or website. These are third party services that allow Second Nature to collect information from you concerning your use of the System, including but not limited to pages visited, links clicked, non-sensitive text entered, mouse movements, and usage of our iPhone and Android apps. These services are used to help Second Nature enhance or improve the user experience on this website and to perform any other function that Second Nature reasonably believe in good faith is required to protect and ensure the proper functionality and security of this website.
- — Third party medical professionals: with additional permission that we will separately explain to you and request your consent for, we may disclose your personal information to a third-party medical professional nominated by you: e.g. your GP or local NHS service. This may be in the form of a discharge letter or an electronic disclosure to an electronic patient record.
- — Threat to health or safety: We may use and disclose your personal information when necessary to prevent a serious threat to your health and safety or the health and safety of the public or another person. Any disclosure, however, would only be to someone able to help prevent the threat.
- — As required by law: Certain laws permit or require certain uses and disclosures of personal information, for example, for public health activities, health oversight activities and law enforcement. We may be required to disclose personal information for these and other compliance purposes, including as may be required by applicable laws and regulations or requested by a judicial process or government agency. In these instances, Second Nature will only use or disclose your personal information to the extent the law requires.
- — For research and publicity purposes: We may use personal information for internal and external research and publicity purposes. This may include publishing aggregate, anonymous information about our users in the context of providing public information and conducting academic research.
- — Transfer of business assets: If Second Nature or substantially all of its assets are acquired by a third party, personal information held by it about its customers will be one of the transferred assets.

National personal information opt-out policy: as part as our contracts with the NHS, we collect, process and disclose confidential patient information. We always make sure that we do so for individual care purposes only; we only use and/or disclose anonymised personal information for research purposes, and we don’t use or disclose confidential patient personal information for planning purposes. As such, we are compliant with the National data opt-out policy.

Except as described above, we will never share your personal information with any other party without your consent.

1. **Legal Bases for Processing European Personal Information**

If you are located in the European Economic Area or the United Kingdom, we only process your personal information when we have a valid “legal basis”, including when:

- — Consent. You have consented to the use of your personal information, for example to send you marketing communications or to use cookies.
- — Contractual necessity. We need your personal information to provide you with the services, for example to respond to your inquiries.
- — Compliance with a legal obligation. We have a legal obligation to use your personal information, for example to comply with tax and accounting obligations.
- — Legitimate interests. We or a third party have a legitimate interest in using your personal information. In particular, we have a legitimate interest in using your personal information for product development and internal analytics purposes, and otherwise to improve the safety, security, and performance of our services. We only rely on our or a third party's legitimate interests to process your personal information when these interests are not overridden by your rights and interests.

1. **Where we store your personal information**

All personal information you provide to us is stored on secure servers with trusted 3rd party suppliers, Amazon Web Services ('AWS') within the European Economic Area ('EEA'). AWS complies with the GDPR and the UK GDPR, which set out several data protection requirements, which apply when personal information is being processed. AWS are industry leaders in the provision of hosting services and take security very seriously - you can find out more about their security policies and processes in their [Security Whitepaper](https://d0.awsstatic.com/whitepapers/aws-security-whitepaper.pdf).

We may transfer personal information outside the EEA or the UK to countries deemed adequate by the European Commission; based on Standard Contractual Clauses; to perform the services that you have requested from us, or with your consent.

Unfortunately, despite these measures, the transmission of information via the internet is never completely secure. Although we will do our best to protect your personal information, we cannot guarantee the security of your information transmitted to the System, and any transmission is at your own risk. Once we have received your personal information, we will use strict procedures to try to prevent unauthorized access in accordance with our Company data protection policy and code of practice, and responsibilities as a registered Data Controller in the UK.

1. **Your rights regarding your personal information**

You have certain rights with respect to your personal information. If we do not agree to a request by you with respect to your personal information, please consult the Second Nature Privacy and Security Officer whose contact information is below.

If you are based in the UK or the European Economic Area and we do not comply with any of the below, you have the right to complain to the ICO or to your local Supervisory Authority, and to a judicial remedy.

- — Restrictions: You have the right to request in writing that we do not disclose certain information about you. To request a restriction, please contact the Privacy and Security Officer whose contact information is below.
- — Confidential Communications: You have the right to request in writing that we restrict the way in which we communicate information regarding your health and health care services, such as ceasing to send email or SMS messages to notify or remind you about aspects of the System or your progress through the Second Nature program. We will make every effort to accommodate your request.
- — Access: You have the right to inspect and copy your personal information maintained by us. Normally, we will provide you with access within one month of your request. To request your personal information:
  - — Please download a subject access request form [here](https://docs.google.com/document/d/19e6SQwJq6e7OYcOETXRyWWvyljdvtuzf/), fill it in and return it to us using the contact details on the form, along with copies of information that confirms your identity (if applicable). Please do not send in any original copies of documents. More details of acceptable types of identification documents are included in the application form.
  - — You can email your completed form and electronic copies of your identification documents to: support@secondnature.io
  - — Or print the form, fill it in and post it with paper copies of your identification documents to:
  - — Second Nature
  - — Scale Space White City
  - — 58 Wood Ln
  - — London, W12 7RZ
  - — United Kingdom
  - — We will endeavour to respond promptly and in any event within one month. You can alternatively call us on +44 20 3488 0769
- — Deletion: You have the right to ask that we delete all information that the System has collected on you via email to the Second Nature privacy and Security whose contact information is below.
- — Amendment: You have the right to request that we amend your written personal information. For instance, you can request that we correct an incorrect date of birth in your records. We will amend your personal information within one month of your request, and will notify you when we have amended your personal information. We can deny your request in certain circumstances, such as when we believe that your personal information is accurate and complete.
- — Personal Information Portability: You have the right to obtain and reuse your personal information from Second Nature for your own purposes across different services. This can be freely downloaded in .csv format within the settings page of your Second Nature account.
- — Objection: You have the right to object to processing based on legitimate interests or the performance of a task in the public interest, to direct marketing, and to processing for the purposes of scientific research & statistics. To request an objection, please contact the Privacy and Security Officer whose contact information is below.
- — Automated Decision Making & Profiling: You have the right not to be subject to a decision based on automated processing and it produces a legal effect or a similarly significant effect on you. To request an opt-out of automated decision making & profiling, please contact the Privacy and Security Officer whose contact information is below.

Before meeting your request, we may ask you to provide reasonable information to verify your identity. Please note that there are exceptions and limitations to each of these rights, and that while any changes you make will be reflected in active user databases instantly or within a reasonable period of time, we may retain information for backups, archiving, prevention of fraud and abuse, analytics, satisfaction of legal obligations, or where we otherwise reasonably believe that we have a legitimate reason to do so.

1. **Personal Information Retention**

As per the ICO's 'Principle 5' and the article 5 of GDPR and the UK GDPR, we retain personal information no longer than is necessary for the purpose we obtained it for. With the context that your personal information may be used for research purposes (as covered in section 3), Second Nature will retain any information held on an individual for up to 10 years after that individual has ceased use of the System. At that point, the individual's information will be deleted. As covered in section 5, you may request that we delete your personal information at any time.

1. **EU Representative**

If you are based in the EU Second Nature Healthy Habits Ltd has appointed DataRep as its Data Protection Representative for the purposes of GDPR, so that you can contact them directly in your home country. DataRep has locations in each of the 27 countries and Norway & Iceland in the European Economic Area (EEA). If you want to raise a question to Second Nature Healthy Habits Ltd, or exercise your rights (explained above) in respect of your personal information, you may do so by:

- — Sending an email to DataRep at datarequest@datarep.com, quoting Second Nature Health Ltd in the subject line.
- — Contacting DataRep using their online webform at www.datarep.com/data-request or mailing your enquiry to DataRep at the most convenient of the addresses that you can find below
  - — Austria: DataRep, City Tower, Brückenkopfgasse 1/6. Stock, Graz, 8020, Austria
  - — Belgium: DataRep, Place de L'Université 16, Louvain-La-Neuve, Waals Brabant, 1348, Belgium
  - — Bulgaria: DataRep, 132 Mimi Balkanska Str., Sofia, 1540, Bulgaria
  - — Croatia: DataRep, Ground & 9th Floor, Hoto Tower, Savska cesta 32, Zagreb, 10000, Croatia
  - — Cyprus: DataRep, Victory House, 205 Archbishop Makarios Avenue, Limassol, 3030, Cyprus
  - — Czech: Republic DataRep, IQ Ostrava Ground floor, 28. rijna 3346/91, Ostrava-mesto, Moravska, Ostrava, Czech Republic
  - — Denmark: DataRep, Lautruphøj 1-3, Ballerup, 2750, Denmark
  - — Estonia: DataRep, 2nd Floor, Tornimae 5, Tallinn, 10145, Estonia
  - — Finland: DataRep, Luna House, 5.krs, Mannerheimintie 12 B, Helsinki, 00100, Finland
  - — France: DataRep, 72 rue de Lessard, Rouen, 76100, France
  - — Germany: DataRep, 3rd and 4th floor, Altmarkt 10 B/D, Dresden, 01067, Germany
  - — Greece: DataRep, 24 Lagoumitzi str, Athens, 17671, Greece
  - — Hungary: DataRep, President Centre, Kálmán Imre utca 1, Budapest, 1054, Hungary
  - — Iceland: DataRep, Kalkofnsvegur 2, 101 Reykjavík, Iceland
  - — Ireland: DataRep, The Cube, Monahan Road, Cork, T12 H1XY, Republic of Ireland
  - — Italy: DataRep, BPM 335368, Via Roma 12, 10073 , Ciriè TO, Italy
  - — Latvia: DataRep, 4th & 5th floors, 14 Terbatas Street, Riga, LV-1011, Latvia
  - — Liechtenstein: DataRep, City Tower, Brückenkopfgasse 1/6. Stock, Graz, 8020, Austria
  - — Lithuania: DataRep, 44A Gedimino Avenue, 01110 Vilnius, Lithuania
  - — Luxembourg: DataRep, BPM 335368, Banzelt 4 A, 6921, Roodt-sur-Syre, Luxembourg
  - — Malta: DataRep, Tower Business Centre, 2nd floor, Tower Street, Swatar, BKR4013, Malta
  - — Netherlands: DataRep, Cuserstraat 93, Floor 2 and 3, Amsterdam, 1081 CN, Netherlands
  - — Norway: DataRep, C.J. Hambros Plass 2c, Oslo, 0164, Norway
  - — Poland: DataRep, Budynek Fronton ul Kamienna 21, Krakow, 31-403, Poland
  - — Portugal: DataRep, Torre de Monsanto, Rua Afonso Praça 30, 7th floor, Algès, Lisbon, 1495-061, Portugal
  - — Romania: "DataRep, 15 Piaţa Charles de Gaulle, nr. 1-T, Bucureşti, Sectorul 1, 011857,
  - — Slovakia: DataRep, Apollo Business Centre II, Block E / 9th floor, 4D Prievozska, Bratislava, 821 09, Slovakia
  - — Slovenia: DataRep, Trg. Republike 3, Floor 3, Ljubljana, 1000, Slovenia
  - — Spain: DataRep, BPM 335368, Avd. Castilla La Mancha Nº 70-1 (Nave A), 45270, Mocejon-Toledo, Spain
  - — Sweden: DataRep, S:t Johannesgatan 2, 4th floor, Malmo, SE - 211 46, Sweden

When mailing enquiries, please mark your letters for “DataRep” and not “Second Nature Healthy Habits Ltd”, otherwise the letter may not reach DataRep. Please refer clearly to Second Nature Healthy Habits Ltd in your correspondence. On receiving your correspondence, we are likely to request evidence of your identity, to ensure your personal data and information connected with it is not provided to anyone other than you.

If you have any concerns over how DataRep will handle the personal data they will require to undertake their services, please refer to their privacy policy at www.datarep.com/privacy-policy.

1. **Children's Privacy**

We do not knowingly collect, maintain, or use personal information from children under 13 years of age, and no part of our Service is directed to children. If you learn that a child has provided us with personal information in violation of this Privacy Policy, then you may alert us at [support@secondnature.io](mailto:support@secondnature.io).

1. **Data Security**

We make reasonable efforts to protect your information by using physical and electronic safeguards designed to improve the security of the information we maintain. However, as no electronic transmission or storage of information can be entirely secure, we can make no guarantees as to the security or privacy of your information.

Second Nature provides the System to referrals provided through the NHS. As such, we are compliant with the Data Security and Protection Toolkit 2019 / 2020, our organisation code is 8JF17.

1. **Concerns or complaints**

If you believe that any of your rights with respect to your personal information has been violated by us, our employees or agents, please communicate with the Second Nature Privacy and Security Officer at: support@secondnature.io for UK users, or DataRep for EU-based users (contact details above)

1. **Amending this Policy**

We reserve the right to revise this Policy and to make the revised Policy effective for all personal information that we created or received prior to the effective date of the revised Policy. If you are a registered user, we will notify you of changes by the email address we have for you on file.

Questions relating to revisions to this Policy may be addressed to the Privacy and Security Officer whose contact information is above. This Policy will be promptly revised if there is a material change to a policy described herein.

Effective Date: This Policy is effective as of May 19th 2021.
