# Supplementary material for: The Association Between Goal Setting and Weight Loss: Prospective Analysis of a Community Weight Loss Program
Source: J Med Internet Res. 2023 Jul 5;25:e43869. doi: 10.2196/43869 (PMC10357317; doi:10.2196/43869)
Supplement: Multimedia Appendix 2 [file jmir_v25i1e43869_app2.docx]

**MULTIMEDIA APPENDIX - TABLES S1-S14**

**The Association Between Goal Setting and Weight Loss: Prospective Analysis of a Community Weight Loss Program**

This is a Multimedia Appendix to a full manuscript published in the J Med Internet Res. For full copyright and citation information see <http://dx.doi.org/10.2196/jmir.43869>

**Table S1: Cross-tabulation analysis to explore the relationship between motivation and goal preference.** Numbers are the percentage in each category.

|  |  | **Motivation** | | | | **Row Total (%)** |
| --- | --- | --- | --- | --- | --- | --- |
|  |  | Appearance | Fitness | Health | Self-efficacy |  |
| **Goal preference** | Low | 34.6 | 15.4 | 30.6 | 19.3 | 15.3 |
|  | Medium | 39.8 | 14.4 | 28.4 | 17.4 | 59.7 |
|  | High | 43.6 | 11.5 | 23.3 | 21.6 | 16.7 |
|  | Undefined | 44.2 | 11.4 | 25.8 | 18.5 | 8.3 |
| **Column Total (%)** | | 40.1 | 13.8 | 27.6 | 18.5 | 100 |

**Table S2: Cross-tabulation analysis to explore the relationship between goal preference and percentage weight loss goal.** Numbers are the percentage in each category.

|  |  | **Goal Preference** | | | | **Row Total (%)** |
| --- | --- | --- | --- | --- | --- | --- |
|  |  | Low | Medium | High | Undefined |  |
| **Percentage weight**  **loss goal** | <5 | 27.0 | 60.2 | 10.5 | 2.2 | 6.5 |
|  | 5-10 | 12.5 | 57.6 | 20.4 | 9.5 | 64.2 |
|  | >10 | 15.3 | 60.6 | 15.8 | 8.3 | 29.3 |
| **Column Total (%)** | | 15.3 | 59.7 | 16.7 | 8.3 | 100 |

**Table S3: Cross-tabulation analysis to explore the relationship between percentage weight loss goal and motivation.** Numbers are the percentage in each category.

|  |  | **Percentage weight loss goal** | | | **Row Total (%)** |
| --- | --- | --- | --- | --- | --- |
|  |  | <5 | 5-10 | >10 |  |
| **Motivation** | Appearance | 6.9 | 62.7 | 30.4 | 40.1 |
|  | Fitness | 8.4 | 62.9 | 28.7 | 13.8 |
|  | Health | 5.2 | 66.7 | 28.1 | 27.6 |
|  | Self-efficacy | 5.9 | 64.8 | 29.3 | 18.5 |
| **Column Total (%)** | | 6.5 | 64.2 | 29.3 | 100 |

**Table S4: Baseline characteristics and weight change categorised by weight loss motivation.**

|  | **Appearance** | **Fitness** | **Health** | **Self-efficacy** | ***P*** ^a^ |
| --- | --- | --- | --- | --- | --- |
|  | (n=14,736) | (n=5,092) | (n=10,177) | (n=6,789) |  |
|  |  |  |  |  |  |
| **Age (years),**  **mean (SD)** | 44.8 (10.8) | 49.2 (11.2) | 49.4 (10.9) | 45.1 (10.7) | <.001 |
| **Sex, n (%)** |  |  |  |  |  |
| Male | 1051 (7.1) | 484 (9.5) | 1046 (10.3) | 311 (4.6) | <.001 |
| Female | 13685 (92.9) | 4608 (90.5) | 9131 (89.7) | 6478 (95.4) |  |
| **BMI (kg/m^2^), mean (SD)** | 31.7 (4.91) | 33.7 (6.26) | 36.8 (7.08) | 35.1 (6.63) | <.001 |
| **IMD Decile, n (%)** |  |  |  |  |  |
| 1-3 | 1659 (11.3) | 659 (12.9) | 1209 (11.9) | 852 (12.5) | <.001 |
| 4-7 | 4819 (32.7) | 1638 (32.2) | 3434 (33.7) | 2200 (32.4) |  |
| 8-10 | 4928 (33.4) | 1525 (29.9) | 3266 (32.1) | 2202 (32.4) |  |
| Missing | 3330 (22.6) | 1270 (24.9) | 2268 (22.3) | 1535 (22.6) |  |
| **Percentage weight loss goal, mean (SD)** | 9.22 (3.98) | 8.97 (10.9) | 9.41 (3.95) | 9.35 (3.88) | <.001 |
| **Weight change at 4 weeks (kg)** |  |  |  |  |  |
| Mean (SD) | -3.88 (2.58) | -4.13 (2.83) | -4.57 (3.37) | -4.15 (2.98) | <.001 |
| Missing, n (%) | 8431 (57.2) | 2957 (58.1) | 5805 (57.0) | 4146 (61.1) |  |
| **Weight change at 12 weeks (kg)** |  |  |  |  |  |
| Mean (SD) | -5.53 (3.87) | -5.98 (4.26) | -6.66 (4.72) | -5.59 (4.48) | <.001 |
| Missing, n (%) | 11632 (78.9) | 4062 (79.8) | 7925 (77.9) | 5548 (81.7) |  |
| **Weight change at 24 weeks (kg)** |  |  |  |  |  |
| Mean (SD) | -5.91 (5.38) | -6.24 (5.65) | -7.40 (6.53) | -5.90 (6.37) | <.001 |
| Missing, n (%) | 12768 (86.6) | 4446 (87.3) | 8746 (85.9) | 6016 (88.6) |  |

^a^*P* values were calculated using t-tests for continuous variables or chi-squared tests for categorical variables.

**Table S5: Baseline characteristics and weight change categorised by goal preference.**

|  | **Low** | **Medium** | **High** | **Undefined** | ***P*** ^a^ |
| --- | --- | --- | --- | --- | --- |
|  | (n=5,597) | (n=21,963) | (n=6,180) | (n=3,054) |  |
|  |  |  |  |  |  |
| **Age (years), mean (SD)** | 47.9 (11.2) | 47.7 (11.0) | 43.8 (10.5) | 43.9 (11.2) | <.001 |
| **Sex, n (%)** |  |  |  |  |  |
| Male | 184 (3.3) | 1502 (6.8) | 816 (13.2) | 390 (12.8) | <.001 |
| Female | 5413(96.7) | 20461(93.2) | 5364 (86.8) | 2664 (87.2) |  |
| **BMI (kg/m^2^), mean (SD)** | 35.5 (6.90) | 33.8 (6.34) | 33.6 (6.34) | 33.6 (6.39) | <.001 |
| **IMD Decile, n (%)** |  |  |  |  |  |
| 1-3 | 710 (12.7) | 2614 (11.9) | 721 (11.7) | 334 (10.9) | .27 |
| 4-7 | 1822 (32.6) | 7251 (33.0) | 2042 (33.0) | 976 (32.0) |  |
| 8-10 | 1750 (31.3) | 7199 (32.8) | 2049 (33.2) | 923 (30.2) |  |
| Missing | 1315 (23.5) | 4899 (22.3) | 1368 (22.1) | 821 (26.9) |  |
| **Percentage weight loss goal, mean (SD)** | 8.76 (4.18) | 9.18 (6.17) | 9.74 (4.38) | 9.75 (3.79) | <.001 |
| **Weight change at 4 weeks (kg)** |  |  |  |  |  |
| Mean (SD) | -4.08 (3.29) | -4.10 (2.80) | -4.45 (3.10) | -4.18 (2.91) | <.001 |
| Missing, n (%) | 3337 (59.6) | 12405 (56.5) | 3845 (62.2) | 1752 (57.4) |  |
| **Weight change at 12 weeks (kg)** |  |  |  |  |  |
| Mean (SD) | -5.77 (4.18) | -5.95 (4.17) | -5.85 (4.74) | -6.22 (4.78) | .17 |
| Missing, n (%) | 4468 (79.8) | 17238 (78.5) | 5101 (82.5) | 2360 (77.3) |  |
| **Weight change at 24 weeks (kg)** |  |  |  |  |  |
| Mean (SD) | -6.34 (6.04) | -6.40 (5.76) | -6.08 (6.37) | -6.97 (6.58) | .11 |
| Missing, n (%) | 4872 (87.0) | 19035 (86.7) | 5440 (88.0) | 2629 (86.1) |  |

^a^*P* values were calculated using t-tests for continuous variables or chi-squared tests for categorical variables.

**Table S6: Baseline characteristics and weight change categorised by percentage weight loss goal.**

|  | **<5** | **5-10** | **>10** | ***P*** ^a^ |
| --- | --- | --- | --- | --- |
|  | (N=2370) | (N=23629) | (N=10795) |  |
|  |  |  |  |  |
| **Age (years), mean (SD)** | 49.0 (12.6) | 46.6 (11.1) | 46.4 (10.8) | <.001 |
| **Sex, n (%)** |  |  |  |  |
| Male | 169 (7.1) | 1810 (7.7) | 913 (8.5) | .04 |
| Female | 2201 (92.9) | 21819 (92.3) | 9882 (91.5) |  |
| **BMI (kg/m^2^), mean (SD)** | 31.4 (5.51) | 34.3 (6.58) | 33.9 (6.24) | <.001 |
| **IMD Decile, n (%)** |  |  |  |  |
| 1-3 | 275 (11.6) | 2858 (12.1) | 1246 (11.5) | .43 |
| 4-7 | 730 (30.8) | 7697 (32.6) | 3664 (33.9) |  |
| 8-10 | 751 (31.7) | 7596 (32.1) | 3574 (33.1) |  |
| Missing | 614 (25.9) | 5478 (23.2) | 2311 (21.4) |  |
| **Weight change at 4 weeks (kg)** |  |  |  |  |
| Mean (SD) | -3.12 (3.05) | -3.33 (2.16) | -5.36 (3.39) | <.001 |
| Missing, n (%) | 1971 (83.2) | 14932 (63.2) | 4436 (41.1) |  |
| **Weight change at 12 weeks (kg)** |  |  |  |  |
| Mean (SD) | -3.86 (4.84) | -3.96 (3.09) | -7.71 (4.41) | <.001 |
| Missing, n (%) | 2166 (91.4) | 20223 (85.6) | 6778 (62.8) |  |
| **Weight change at 24 weeks (kg)** |  |  |  |  |
| Mean (SD) | -3.06 (4.52) | -3.45 (4.28) | -8.65 (6.08) | <.001 |
| Missing, n (%) | 2230 (94.1) | 21692 (91.8) | 8054 (74.6) |  |

^a^*P* values were calculated using t-tests for continuous variables or chi-squared tests for categorical variables.

**Table S7: Results of mixed effects model for the association between weight loss motivation and weight using all available data (n = 28,391) or completers (n = 2,514).**^a^

| **Variables** | ***All Data (n = 28,391)*** | | | **Completers (n = 2,514)** | | |
| --- | --- | --- | --- | --- | --- | --- |
|  | **Coef** | **95% CI** | ***P*** | **Coef** | **95% CI** | ***P*** |
| **Motivations**  **(ref = Appearance)** |  |  |  |  |  |  |
| Fitness | 6.06 | (5.41,6.71) | <.001 | 6.37 | (4.33,8.4) | <.001 |
| Health | 14.81 | (14.29,15.33) | <.001 | 14.71 | (13.09,16.33) | <.001 |
| Self-efficacy | 10.08 | (9.51,10.66) | <.001 | 8.42 | (6.43,10.4) | <.001 |
|  |  |  |  |  |  |  |
| **Week (ref = 0)** |  |  |  |  |  |  |
| 4 | -3.80 | (-3.91,-3.7) | <.001 | -4.59 | (-4.84,-4.35) | <.001 |
| 12 | -5.24 | (-5.38,-5.11) | <.001 | -6.33 | (-6.57,-6.08) | <.001 |
| 24 | -5.57 | (-5.73,-5.4) | <.001 | -6.77 | (-7.01,-6.52) | <.001 |
|  |  |  |  |  |  |  |
| **Interaction terms** |  |  |  |  |  |  |
| Fitness*week4 | -0.22 | (-0.42,-0.02) | .03 | -0.12 | (-0.59,0.36) | .64 |
| Fitness*week12 | -0.41 | (-0.68,-0.14) | <.001 | -0.26 | (-0.74,0.22) | .28 |
| Fitness*week24 | -0.38 | (-0.7,-0.05) | .03 | -0.34 | (-0.81,0.14) | .17 |
| Health*week4 | -0.73 | (-0.89,-0.58) | <.001 | -0.75 | (-1.12,-0.38) | <.001 |
| Health*week12 | -1.12 | (-1.32,-0.91) | <.001 | -1.15 | (-1.52,-0.79) | <.001 |
| Health*week24 | -1.40 | (-1.65,-1.15) | <.001 | -1.54 | (-1.91,-1.17) | <.001 |
| Self-efficacy*week4 | -0.25 | (-0.44,-0.07) | .01 | -0.14 | (-0.61,0.32) | .55 |
| Self-efficacy*week12 | -0.06 | (-0.31,0.19) | .63 | 0.21 | (-0.25,0.68) | .37 |
| Self-efficacy*week24 | 0.08 | (-0.22,0.39) | .59 | 0.21 | (-0.25,0.68) | .37 |
| ^a^ Models were adjusted for sex, age, IMD decile, type 2 or pre-diabetes. 8,403 participants had missing IMD values. | | | | | | |

**Table S8: Results of mixed effects model for the association between goal preference and weight using all available data (n = 26,158) or completers (n = 2,294).^a^**

| **Variables** | **All Data (n = 26,158)** | | | **Completers (n = 2,294)** | | |
| --- | --- | --- | --- | --- | --- | --- |
|  | **Coef** | **95% CI** | ***P*** | **Coef** | **95% CI** | ***P*** |
| **Goal Preference**  **(ref = medium)** |  |  |  |  |  |  |
| High | -0.01 | (-0.61,0.59) | .98 | -0.24 | (-2.31,1.83) | .82 |
| Low | 3.60 | (2.98,4.22) | <.001 | 3.32 | (1.34,5.3) | <.001 |
|  |  |  |  |  |  |  |
| **Week (ref = 0)** |  |  |  |  |  |  |
| 4 | -4.03 | (-4.11,-3.95) | <.001 | -4.77 | (-4.96,-4.58) | <.001 |
| 12 | -5.63 | (-5.74,-5.53) | <.001 | -6.63 | (-6.82,-6.43) | <.001 |
| 24 | -6.02 | (-6.16,-5.89) | <.001 | -7.23 | (-7.42,-7.03) | <.001 |
|  |  |  |  |  |  |  |
| **Interaction terms** |  |  |  |  |  |  |
| High*Week4 | -0.29 | (-0.47,-0.1) | <.001 | -0.25 | (-0.7,0.21) | .29 |
| High*Week12 | 0.06 | (-0.19,0.31) | .63 | 0.003 | (-0.45,0.46) | .99 |
| High*Week24 | 0.34 | (0.05,0.64) | .02 | 0.19 | (-0.26,0.65) | .41 |
| Low*Week4 | -0.02 | (-0.21,0.16) | .79 | -0.10 | (-0.53,0.34) | .67 |
| Low*Week12 | 0.11 | (-0.14,0.36) | .38 | -0.01 | (-0.44,0.43) | .98 |
| Low*Week24 | -0.01 | (-0.31,0.29) | .95 | 0.02 | (-0.41,0.46) | .92 |
| ^a^ Models were adjusted for sex, age, IMD decile, type 2 or pre-diabetes. 8,403 participants had missing IMD values. 3,054 participants had missing goal preference values. | | | | | | |

**Table S9: Results of mixed effects model for the association between percentage weight loss goal and weight using all available data (n = 28,391) or completers (n = 2,514).^a^**

| **Variables** | **All Data (n = 28,391)** | | | **Completers (n = 2,514)** | | |
| --- | --- | --- | --- | --- | --- | --- |
|  | **Coef** | **95% CI** | ***P*** | **Coef** | **95% CI** | ***P*** |
| **Percentage category**  **(ref = 5-10%)** |  |  |  |  |  |  |
| <5% | -7.99 | (-8.9,-7.09) | <.001 | -2.13 | (-6.49,2.24) | .34 |
| >10% | -0.99 | (-1.47,-0.51) | <.001 | 0.35 | (-1.1,1.8) | .64 |
|  |  |  |  |  |  |  |
| **Week (ref = 0)** |  |  |  |  |  |  |
| 4 | -3.28 | (-3.36,-3.2) | <.001 | -3.85 | (-4.09,-3.62) | <.001 |
| 12 | -3.83 | (-3.94,-3.71) | <.001 | -4.54 | (-4.78,-4.3) | <.001 |
| 24 | -3.27 | (-3.42,-3.12) | <.001 | -4.18 | (-4.42,-3.94) | <.001 |
|  |  |  |  |  |  |  |
| **Interaction terms** |  |  |  |  |  |  |
| <5*Week4 | 0.37 | (-0.01,0.75) | .05 | -0.18 | (-1.08,0.73) | .70 |
| <5*Week12 | 0.43 | (-0.08,0.93) | .10 | -0.33 | (-1.24,0.57) | .47 |
| <5*Week24 | 0.59 | (0.0,1.18) | .05 | -0.14 | (-1.04,0.77) | .77 |
| >10*Week4 | -2.01 | (-2.13,-1.88) | <.001 | -1.64 | (-1.94,-1.34) | <.001 |
| >10*Week12 | -3.69 | (-3.86,-3.53) | <.001 | -3.50 | (-3.8,-3.2) | <.001 |
| >10*Week24 | -5.21 | (-5.41,-5.01) | <.001 | -5.04 | (-5.34,-4.73) | <.001 |
| ^a^ Models were adjusted for sex, age, IMD decile, type 2 or pre-diabetes. 8,403 participants had missing IMD values. | | | | | | |

**Table S10:** **Results of mixed effects model for the association between weight loss motivation and weight using the missing-indicator method (N = 36,794).^a^**

| **Variables** | **All Data (N = 36,794)** | | |
| --- | --- | --- | --- |
|  | **Coef** | **95% CI** | ***P*** |
| **Motivations (ref = Appearance)** |  |  |  |
| Fitness | 6.06 | (5.49,6.63) | <.001 |
| Health | 14.88 | (14.42,15.34) | <.001 |
| Self-efficacy | 9.88 | (9.38,10.39) | <.001 |
|  |  |  |  |
| **Week (ref = 0)** |  |  |  |
| 4 | -3.80 | (-3.89,-3.71) | <.001 |
| 12 | -5.25 | (-5.37,-5.13) | <.001 |
| 24 | -5.59 | (-5.74,-5.45) | <.001 |
|  |  |  |  |
| **Interaction terms** |  |  |  |
| Fitness*week4 | -0.27 | (-0.45,-0.09) | <.001 |
| Fitness*week12 | -0.44 | (-0.68,-0.2) | <.001 |
| Fitness*week24 | -0.36 | (-0.66,-0.07) | .01 |
| Health*week4 | -0.73 | (-0.87,-0.59) | <.001 |
| Health*week12 | -1.10 | (-1.29,-0.92) | <.001 |
| Health*week24 | -1.42 | (-1.64,-1.19) | <.001 |
| Self-efficacy*week4 | -0.28 | (-0.45,-0.12) | <.001 |
| Self-efficacy*week12 | -0.08 | (-0.3,0.14) | .48 |
| Self-efficacy*week24 | -0.05 | (-0.33,0.22) | .70 |
| ^a^ Models were adjusted for sex, age, IMD decile, type 2 or pre-diabetes. | | | |

**Table S11: Results of mixed effects model for the association between goal preference and weight using the missing-indicator method (N = 36,794).^a^**

| **Variables** | **All Data (N = 36,794)** | | |
| --- | --- | --- | --- |
|  | **Coef** | **95% CI** | ***P*** |
| **Goal Preference (ref = medium)** |  |  |  |
| High | -0.18 | (-0.71,0.35) | .51 |
| Low | 3.73 | (3.19,4.28) | <.001 |
| Missing | -0.51 | (-1.22,0.19) | .15 |
|  |  |  |  |
| **Week (ref = 0)** |  |  |  |
| 4 | -4.04 | (-4.11,-3.97) | <.001 |
| 12 | -5.65 | (-5.75,-5.55) | <.001 |
| 24 | -6.08 | (-6.2,-5.96) | <.001 |
|  |  |  |  |
| **Interaction terms** |  |  |  |
| High*Week4 | -0.29 | (-0.46,-0.13) | <.001 |
| High*Week12 | 0.04 | (-0.19,0.27) | .72 |
| High*Week24 | 0.28 | (0.01,0.54) | .04 |
| Low*Week4 | -0.01 | (-0.17,0.16) | .95 |
| Low*Week12 | 0.15 | (-0.08,0.37) | .20 |
| Low*Week24 | 0.07 | (-0.2,0.34) | .59 |
| Missing*Week4 | -0.10 | (-0.31,0.12) | .37 |
| Missing*Week12 | -0.25 | (-0.52,0.03) | .08 |
| Missing*Week24 | -0.42 | (-0.75,-0.08) | .02 |

^a^ Models were adjusted for sex, age, IMD decile, type 2 or pre-diabetes.

**Table S12: Results of mixed effects model for the association between percentage weight loss goal and weight using the missing-indicator method (N = 36,794).^a^**

| **Variables** | **All Data (N = 36,794)** | | |
| --- | --- | --- | --- |
|  | **Coef** | **95% CI** | ***P*** |
| **Percentage category**  **(ref = 5-10%)** |  |  |  |
| <5% | -7.78 | (-8.56,-7) | <.001 |
| >10% | -1.12 | (-1.54,-0.7) | <.001 |
|  |  |  |  |
| **Week (ref = 0)** |  |  |  |
| 4 | -3.29 | (-3.37,-3.22) | <.001 |
| 12 | -3.83 | (-3.94,-3.73) | <.001 |
| 24 | -3.31 | (-3.44,-3.17) | <.001 |
|  |  |  |  |
| **Interaction terms** |  |  |  |
| <5*Week4 | 0.44 | (0.1,0.78) | .01 |
| <5*Week12 | 0.48 | (0.04,0.93) | .03 |
| <5*Week24 | 0.76 | (0.23,1.28) | .01 |
| >10*Week4 | -2.00 | (-2.11,-1.89) | <.001 |
| >10*Week12 | -3.72 | (-3.86,-3.57) | <.001 |
| >10*Week24 | -5.22 | (-5.4,-5.05) | <.001 |

^a^ Models were adjusted for sex, age, IMD decile, type 2 or pre-diabetes.

**Table S13: Mediation analysis results for the association between weight loss motivation and weight, as mediated by total engagement.^a^**

| **Variables** | **Step 1 - Association with weight** | | | **Step 2 - Association with total engagement** | | | **Step 3 - Association with weight**  **adjusted for engagement** | | |
| --- | --- | --- | --- | --- | --- | --- | --- | --- | --- |
|  | **Coef** | **95% CI** | ***P*** | **Coef** | **95% CI** | ***P*** | **Coef** | **95% CI** | ***P*** |
| **Motivation (ref = appearance)** |  |  |  |  |  |  |  |  |  |
| Fitness | 6.06 | (5.41,6.71) | <.001 | -3.55 | (-16.12,9.02) | .58 | 6.06 | (5.41,6.71) | <.001 |
| Health | 14.81 | (14.29,15.33) | <.001 | -1.27 | (-11.24,8.69) | .80 | 14.81 | (14.29,15.33) | <.001 |
| Self-efficacy | 10.08 | (9.51,10.66) | <.001 | 0.69 | (-10.45,11.84) | .90 | 10.08 | (9.51,10.66) | <.001 |
|  |  |  |  |  |  |  |  |  |  |
| **Week (ref = 0)** |  |  |  |  |  |  |  |  |  |
| 4 | -3.80 | (-3.91,-3.7) | <.001 | 200.73 | (194.81,206.66) | <.001 | -3.18 | (-3.28,-3.07) | <.001 |
| 12 | -5.24 | (-5.38,-5.11) | <.001 | 319.49 | (313.56,325.42) | <.001 | -3.96 | (-4.11,-3.81) | <.001 |
| 24 | -5.57 | (-5.73,-5.4) | <.001 | 361.77 | (355.85,367.7) | <.001 | -3.88 | (-4.07,-3.7) | <.001 |
|  |  |  |  |  |  |  |  |  |  |
| **Interaction terms** |  |  |  |  |  |  |  |  |  |
| Fitness*Week4 | -0.22 | (-0.42,-0.02) | .03 | -19.89 | (-31.72,-8.06) | <.001 | -0.24 | (-0.44,-0.05) | .02 |
| Fitness*Week12 | -0.41 | (-0.68,-0.14) | <.001 | -27.63 | (-39.46,-15.8) | <.001 | -0.42 | (-0.69,-0.16) | <.001 |
| Fitness*Week24 | -0.38 | (-0.7,-0.05) | .03 | -27.40 | (-39.23,-15.57) | <.001 | -0.36 | (-0.68,-0.04) | .03 |
| Health*Week4 | -0.73 | (-0.89,-0.58) | <.001 | -10.55 | (-19.81,-1.29) | .03 | -0.77 | (-0.92,-0.62) | <.001 |
| Health*Week12 | -1.12 | (-1.32,-0.91) | <.001 | -12.17 | (-21.44,-2.91) | .01 | -1.15 | (-1.35,-0.95) | <.001 |
| Health*Week24 | -1.40 | (-1.65,-1.15) | <.001 | -6.44 | (-15.7,2.82) | .17 | -1.41 | (-1.65,-1.16) | <.001 |
| Self-efficacy*Week4 | -0.25 | (-0.44,-0.07) | .01 | -13.83 | (-24.39,-3.28) | .01 | -0.27 | (-0.45,-0.09) | <.001 |
| Self-efficacy*Week12 | -0.06 | (-0.31,0.19) | .63 | -34.36 | (-44.92,-23.81) | <.001 | -0.14 | (-0.38,0.11) | .26 |
| Self-efficacy*Week24 | 0.08 | (-0.22,0.39) | .59 | -39.88 | (-50.43,-29.33) | <.001 | -0.05 | (-0.35,0.25) | .75 |
|  |  |  |  |  |  |  |  |  |  |
| **Total engagement**  **(per 100 engagements)** |  |  |  |  |  |  | -0.21 | (-0.22,-0.19) | <.001 |

^a^Models were adjusted for sex, age, IMD decile, type 2 or pre-diabetes.

**Table S14: Mediation analysis results for the association between percentage weight loss goal and weight, as mediated by total engagement.^a^**

| **Variables** | **Step 1 - Association with weight** | | | **Step 2 - Association with total engagement** | | | **Step 3 - Association with weight**  **adjusted for engagement** | | |
| --- | --- | --- | --- | --- | --- | --- | --- | --- | --- |
|  | **Coef** | **95% CI** | **P** | **Coef** | **95% CI** | **P** | **Coef** | **95% CI** | **P** |
| **Percentage category (ref = 5-10%)** |  |  |  |  |  |  |  |  |  |
| <5% | -7.99 | (-8.9,-7.09) | <.001 | -3.44 | (-19.59,12.72) | .68 | -7.99 | (-8.9,-7.09) | <.001 |
| >10% | -0.99 | (-1.47,-0.51) | <.001 | 0.01 | (-8.49,8.51) | 0.998 | -0.99 | (-1.47,-0.51) | <.001 |
|  |  |  |  |  |  |  |  |  |  |
| **Week (ref = 0)** |  |  |  |  |  |  |  |  |  |
| 4 | -3.28 | (-3.36,-3.2) | <.001 | 169.66 | (165.11,174.22) | <.001 | -2.83 | (-2.92,-2.75) | <.001 |
| 12 | -3.83 | (-3.94,-3.71) | <.001 | 255.55 | (251.0,260.1) | <.001 | -2.95 | (-3.07,-2.82) | <.001 |
| 24 | -3.27 | (-3.42,-3.12) | <.001 | 280.64 | (276.09,285.19) | <.001 | -2.17 | (-2.33,-2.00) | <.001 |
|  |  |  |  |  |  |  |  |  |  |
| **Interaction terms** |  |  |  |  |  |  |  |  |  |
| <5*Week4 | 0.37 | (-0.01,0.75) | .05 | -96.48 | (-111.8,-81.15) | <.001 | 0.31 | (-0.06,0.68) | .11 |
| <5*Week12 | 0.43 | (-0.08,0.93) | .10 | -139.85 | (-155.17,-124.52) | <.001 | 0.34 | (-0.15,0.84) | .17 |
| <5*Week24 | 0.59 | (0,1.18) | .05 | -147.50 | (-162.82,-132.18) | <.001 | 0.51 | (-0.07,1.09) | .09 |
| >10*Week4 | -2.01 | (-2.13,-1.88) | <.001 | 96.58 | (88.52,104.64) | <.001 | -1.93 | (-2.05,-1.81) | <.001 |
| >10*Week12 | -3.69 | (-3.86,-3.53) | <.001 | 197.83 | (189.77,205.9) | <.001 | -3.49 | (-3.65,-3.33) | <.001 |
| >10*Week24 | -5.21 | (-5.41,-5.01) | <.001 | 258.99 | (250.93,267.06) | <.001 | -4.84 | (-5.04,-4.64) | <.001 |
|  |  |  |  |  |  |  |  |  |  |
| **Total engagement**  **(per 100 engagements)** |  |  |  |  |  |  | -0.16 | (-0.17,-0.15) | <.001 |

^a^Models were adjusted for sex, age, IMD decile, type 2 or pre-diabetes
