# Supplementary material for: The Association Between Goal Setting and Weight Loss: Prospective Analysis of a Community Weight Loss Program
Source: J Med Internet Res. 2023 Jul 5;25:e43869. doi: 10.2196/43869 (PMC10357317; doi:10.2196/43869)
Supplement: Multimedia Appendix 3 [file jmir_v25i1e43869_app3.docx]

**MULTIMEDIA APPENDIX - FIGURE S1**

**The Association Between Goal Setting and Weight Loss: Prospective Analysis of a Community Weight Loss Program**

This is a Multimedia Appendix to a full manuscript published in the J Med Internet Res. For full copyright and citation information see <http://dx.doi.org/10.2196/jmir.43869>


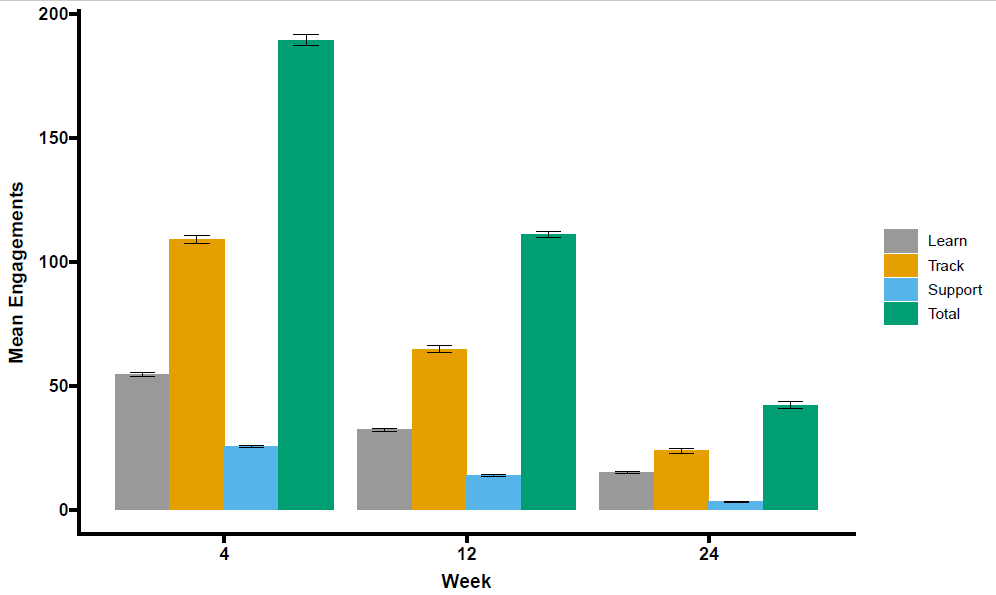


**Figure S1: Mean learn, track, support, and total engagements at 4, 12, and 24 weeks.** Values are mean engagements from 0-4 weeks, 4-12 weeks, and 12-24 weeks. Errors bars are 95% confidence intervals.
